# 283. Screening the PE/PPE secreted effectors of *Mycobacterium tuberculosis* to uncover novel virulence-promoting genes

**DOI:** 10.1093/ofid/ofad500.355

**Published:** 2023-11-27

**Authors:** Benjamin Koleske, Jessica Shen, Manish Gupta, William Bishai

**Affiliations:** Johns Hopkins University, Baltimore, Maryland; Johns Hopkins University, Baltimore, Maryland; Johns Hopkins University, Baltimore, Maryland; Johns Hopkins University School of Medicine, Baltimore, Maryland

## Abstract

**Background:**

*Mycobacterium tuberculosis* (M.tb) remains the leading cause of infectious disease mortality, with over 10 million active cases and 1.5 million deaths annually at the global scale. Although M.tb infection prompts highly inflammatory granulomatous lesions in the lung, the bacterium is not eradicated by the immune cell infiltrate. Instead, M.tb uses numerous virulence factors to facilitate growth within macrophages, including secretory proteins that subvert host immune processes. In this work, we examined the substrates of ESX-5, a prolific type VII secretion system unique to slow-growing pathogenic mycobacteria, for their roles in contributing to bacterial virulence in a mouse lung infection model. These secretory substrates, the PE and PPE family proteins (named for conserved Pro-Glu and Pro-Pro-Glu domains), have evolutionarily expanded from 11 members in the harmless commensal *M. smegmatis* to 167 members in the M.tb H37Rv reference strain, suggesting they serve critical functions for an intracellular pathogen with an otherwise reduced genome. Certain PE/PPE genes have been implicated in a broad range of immune relevant functions, including TLR signaling, apoptosis inhibition, and NF-κB activation, though the vast majority remain understudied.

M.tb secretion during infection

**Figure:**
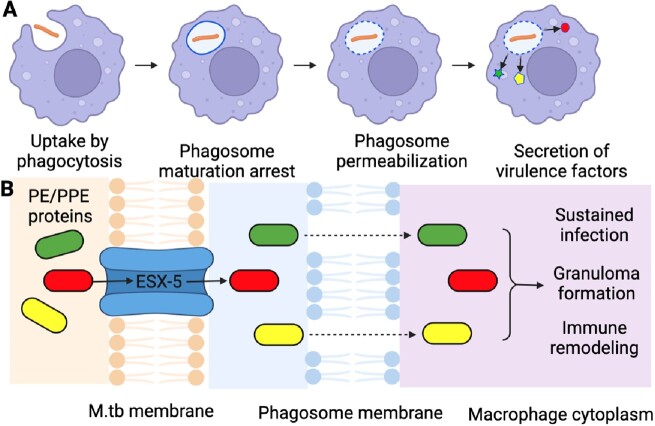

(A) Following phagocytosis by a macrophage, M.tb blocks phagosome maturation and permeabilizes the phagosome to secrete effectors into the macrophage cytoplasm. (B) The ESX-5 system secretes a large (> 150) repertoire of PE/PPE proteins predicted to alter host signaling pathways.

**Methods:**

We performed Tn-Seq in the TB mouse model using a pooled library of 81 M.tb strains with single transposon insertions in known PE/PPE genes, all of which show no *in vitro* growth defect, to identify mutants that demonstrate an *in vivo* fitness cost.

**Results:**

Bacterial genomes were harvested from the lungs of aerosol-infected BALB/c mice at acute (1 week) and subacute (3 week) timepoints and compared to the composition of the initial pool (day 1). Deep sequencing demonstrated significant decreases in the prevalence of 20 strains over time; furthermore, the findings were highly reproducible across all animals (n=10 per timepoint), showed time-dependent kinetics, and identified several PE/PPE genes previously found to contribute to M.tb pathogenesis.

**Conclusion:**

Our mouse Tn-Seq screen of 81 M.tb PE/PPE mutants identified 20 mutants that were attenuated for growth in mice in a pooled competition format. Thus, a high proportion of PE/PPE genes may be virulence-promoting factors.

**Disclosures:**

**All Authors**: No reported disclosures

